# Improving Clinical Patient Activation and Strengthening Health Outcomes: Findings from a Quasi-Experimental Study

**DOI:** 10.3390/jcm15010301

**Published:** 2025-12-31

**Authors:** Saad Mohammad Alsaad, Malak Fahad Almalki, Malak Awadh Alotaibi, Enas Fahad Alaraik, Haytham Ibrahim AlSaif, Abdullah A. Alrasheed, Ali Kerari, Ghareeb Bahari

**Affiliations:** 1 Department of Family and Community Medicine, College of Medicine, King Saud University, Riyadh 12373, Saudi Arabia; salsaad@ksu.edu.sa (S.M.A.); hayalsaif@ksu.edu.sa (H.I.A.); aalrasheed1@ksu.edu.sa (A.A.A.); 2 Prince Faisal Bin Bandar Chair for Geriatric Research, College of Medicine, King Saud University, Riyadh 11451, Saudi Arabia; 3 Medical Physics Section, King Saud University Medical City, Riyadh 12372, Saudi Arabia; malmalki@ksu.edu.sa; 4 Saudi Central Board for Accreditation of Healthcare Institutions, Riyadh 12264, Saudi Arabia; mlalotaibi@cbahi.gov.sa; 5 Family Medicine Center, King Saud University Medical City, King Saud University, Riyadh 12372, Saudi Arabia; enaas-99@hotmail.com; 6 Nursing Administration and Education Department, College of Nursing, King Saud University, Riyadh 11421, Saudi Arabia; alikariri@ksu.edu.sa

**Keywords:** family medicine, patient activation, patient engagement, primary care, Saudi Arabia

## Abstract

**Background/Objectives**: The complexity of healthcare systems and the unclear interactions within them remain key challenges to improving quality and outcomes. The Patient Activation Measure (PAM-13) is a tool that offers insight into patient interactions with healthcare services and has been used for nearly 20 years. However, its application in tertiary healthcare facilities in Saudi Arabia has not been evaluated. This study aimed to assess the feasibility and acceptability of implementing the PAM-13 in a university Family Medicine Center and to evaluate its effect on enhancing patients’ engagement, activation, self-management, and participation in routine care. **Methods**: A quasi-experimental study was conducted from November 2024 to June 2025 using stratified cluster sampling from the diabetes and chronic diseases unit, care of older adults’ unit, and general family medicine unit. The estimated sample size was 65 patients. Statistical analyses were performed using SPSS. **Results**: Baseline PAM-13 scores varied across participants, with most patients in levels 2 and 3. Following the tailored intervention, activation significantly improved in the intervention group (*p* = 0.004), particularly among those initially in levels 1 and 2. Activation scores in the control group declined. No significant short-term changes were observed in clinical outcomes, including blood pressure, glucose, or cholesterol levels. **Conclusions**: Tailored interventions based on PAM-13 enhanced patient activation in a tertiary care setting. Patients with lower baseline activation showed notable improvements in engagement and self-management. Further longitudinal, multicenter studies are needed to determine the sustainability and clinical impact of these improvements.

## 1. Introduction

Patient-centered care has become a core principle in modern healthcare systems, emphasizing respect for patients’ values, needs, and active involvement in their care [1,2,3]. A systematic review of patient-centered care highlighted cultural and organizational barriers that impede effective implementation and underscored the need for context-specific strategies to strengthen patient-centered practices in regional healthcare systems [4]. Patient activation stems from this broader framework, functioning as a measurable indicator of how prepared individuals are to manage their health and engage with healthcare providers [5,6].

Patient activation is critical in shifting from provider-centered to patient-centered care, enabling individuals to become active participants rather than passive recipients. Traditional provider-centered models relied heavily on clinician-directed decision-making, while the growing emphasis on patient-centeredness recognizes that optimal outcomes depend on patients’ engagement, knowledge, and confidence. Higher activation levels are associated with improved self-management, better clinical outcomes, and lower healthcare costs, positioning patient activation as a practical mechanism through which patient-centered care enhances quality, safety, and efficiency [7,8].

Implementation of patient-centered practices has been linked to significant improvements in physical functioning among Accountable Care Organization patients with coexisting physical and mental health conditions. More activated patients report better emotional, physical, and social outcomes [9,10,11]. The importance of patient activation has also been highlighted in recent reform initiatives; for example, the Centre for Medicare and Medicaid Innovation designated patient activation and engagement as evaluation criteria for Pioneer Accountable Care Organization applications [12].

Patient activation is recognized as a patient-reported outcome and a quality-of-care measure in the United States [12]. Researchers found that changes in activation correlated with changes in over 50% of measured outcomes, and associated healthcare costs shifted accordingly [13]. Promoting patient activation may reduce demand for healthcare services, as patients who are invested in self-management demonstrate improved biometrics and more appropriate use of healthcare and emergency services [14,15].

Patient activation interventions have shown meaningful effects on physiological, psychological, behavioral, and health-related quality-of-life outcomes in chronic disease contexts [16]. Evidence indicates that encouraging patient engagement enhances outcomes and well-being across the lifespan and disease spectrum, from prevention to chronic disease management. Advances in recent decades include shared decision-making, promotion of healthy behaviors, and improved chronic disease self-management, all reinforced by the importance of addressing health literacy [17].

A cross-sectional study in Al Madinah, Saudi Arabia, involving 421 patients found that most individuals had low adherence scores on the Morisky Medication Adherence Scale (8-MMAS) and a mean Patient Activation Measure (PAM-13) score of 51.93 (Level 2), indicating limited confidence and knowledge for taking action [18]. Tailoring interventions for patients with type 2 diabetes mellitus (T2DM) based on activation levels improved clinical outcomes and self-management behaviors [19]. A pilot randomized controlled trial showed that adding personalized text messages to a multi-component health program increased patient activation [20].

A randomized primary care trial evaluating a four-session activation program for patients with chronic pain found no increase in activation; however, participants reported reduced depression, improved physical health, and greater adoption of non-pharmacologic pain strategies [21]. An evaluation of the Mayo Clinic’s Adult Medical Care Coordination program demonstrated that coordinated nursing support, including home visits and follow-up calls, resulted in higher activation compared with usual care [22]. A systematic review and meta-analysis reported that digital and online interventions generally enhance activation by supporting self-management and engagement [23,24].

In Saudi Arabia, the Saudi Vision 2030 initiative aims to transform the national healthcare system to improve quality, efficiency, and sustainability. Despite strong global evidence supporting patient activation, research in the Middle East and Gulf regions remains limited. Most existing studies originate from Western contexts, where cultural norms, health literacy, and system structures differ significantly. These differences highlight the need to explore how cultural expectations, systemic characteristics, and socioeconomic factors shape patient activation in Arab societies. Therefore, this study aimed to evaluate the feasibility and acceptability of implementing the PAM-13 in a University Family Medicine Center and to examine its impact on patients’ activation and engagement in healthcare.

## 2. Materials and Methods

### 2.1. Research Design and Setting

A quasi-experimental pretest–post-test intervention design was employed at a Family Medicine Center (FMC) within a University Medical City from November 2024 to June 2025. This approach assessed participants’ outcomes before and after the intervention without random group assignment. Three FMC units were selected for their accessibility: The Diabetes and Chronic Diseases Unit, the Care of the Elderly Unit, and the General Family Medicine Unit. The FMC provides comprehensive primary care, including preventive and acute services, and functions as a teaching site for medical students and family medicine residents.

### 2.2. Sampling Process

A stratified cluster sampling technique ensured representation from varied patient populations. The sample size was determined a priori based on statistical-power considerations for a quasi-experimental pre-test–post-test study design. Using an expected medium effect size (Cohen’s d = 0.50), a two-sided significance level of α = 0.05, and a desired statistical power of 0.80, the minimum required sample size was estimated to be 64. To handle potential attrition or missing data, the sample size was increased by 10%, providing a final sample size of 70 participants to be enrolled. During follow-up, four participants withdrew and one participant passed away, yielding 65 patients for the final analysis. The study population consisted of adults receiving care across the selected FMC units, ensuring heterogeneity in clinical profiles and disease characteristics. Eligible participants were adults (≥18 years) actively receiving care at one of the selected FMC units during the study period, with eligibility operationalized through a review of electronic medical records to confirm age, care site, and at least one documented clinical encounter.

Exclusion criteria were established to maintain the accuracy of PAM-13 responses. Patients were excluded if they had diagnosed mental health disorders or cognitive impairments that could interfere with comprehension or self-assessment. These diagnosed mental-health disorders and cognitive impairment were identified on the basis of documented diagnoses or clinician notes in the electronic medical records. Individuals aged <18 years or >90 years and pregnant individuals were excluded to ensure a clinically comparable adult study population and to minimize variability related to age-related or pregnancy-specific physiological factors.

### 2.3. Instrumentation

Data collection included sociodemographic and clinical variables, along with patient-reported activation and engagement measures. Demographic data such as age, sex, and smoking status were obtained from electronic medical records. Clinical indicators included glucose level, total cholesterol, and systolic and diastolic blood pressure.

The primary instrument for assessing patient activation and engagement was the PAM-13, a validated and widely adopted tool that measures an individual’s knowledge, skills, and confidence in managing their health and healthcare. The PAM-13 was administered as an online self-report questionnaire completed electronically during clinic visits or follow-up sessions. The instrument measures three core dimensions of patient activation: self-reported knowledge, motivation, and self-management skills, collectively reflecting an individual’s capacity to engage effectively in care.

The PAM-13 consists of 13 items rated on a 4-point Likert scale from 1 (“strongly disagree”) to 4 (“strongly agree”). Responses are aggregated into an overall activation score and converted into four standardized activation levels. Items 1–2 correspond to Level 1 (Disengaged and Overwhelmed), items 3–8 to Level 2 (Becoming Aware but Still Struggling), items 9–11 to Level 3 (Taking Action), and items 12–13 to Level 4 (Maintaining Behaviors and Pushing Forward). Higher scores indicate greater activation and stronger engagement in health-related behaviors.

The PAM-13 has demonstrated high reliability across diverse populations, with Cronbach’s α typically ranging from 0.85 to 0.91, as well as strong construct validity in chronic and general patient groups. This study used the authorized Arabic version (PAM-13-A) to ensure linguistic and cultural appropriateness, with permission obtained from Insignia Health, LLC (Portland, OR, USA). The instrument’s design allowed for efficient administration within the clinical workflow while maintaining high response rates and data integrity. PAM level and clinical outcome data were collected at baseline and post-intervention to enable comparison with changes in activation.

### 2.4. Procedures

This study employed a two-group quasi-experimental pre-test–post-test design, including an intervention group and a control group receiving usual care, with assessments conducted at baseline and after 6 months. Interventions provided to the intervention group were tailored according to patients’ activation levels as measured using the PAM-13, according to Insignia Health recommendations and the structured patient education guidelines used in this project. Prior to implementation, physicians and health education specialists received standardized training on the PAM framework and level-specific engagement strategies. The patient education process was guided by the APIE model (Assessment, Planning, Implementation, and Evaluation) [24]. All tailored interventions were delivered through the Health Education Center by healthcare professionals. Data were collected at baseline and at 6 months to assess changes in patient activation and related outcomes, while the active intervention phase was conducted over a 3-month period.

Patients classified as having lower activation (PAM Levels 1 and 2) received tailored educational interventions to address identified knowledge gaps, barriers, and self-management challenges. These interventions consisted of three structured education sessions delivered over 3 months by health education specialists. The first session was conducted face-to-face to allow comprehensive assessment of sociodemographic factors, learning needs, barriers, and patient preferences, followed by two subsequent sessions delivered either in person or virtually based on patient preference. Education content learning, goal setting, and personalized action plan development. Patients in these levels also attended routine follow-up clinic visits every 3 months, supported by four participating physicians, to reinforce learning and monitor progress. Patients with moderate and high activation levels (Level 3 and 4) received routine care and were monitored over time.

Intervention fidelity was ensured by strict adherence to the predefined intervention protocol. The principal investigator and trained research team members consistently applied the APIE framework to guide assessment, planning, implementation, and evaluation across all PAM levels. Documentation was completed after each encounter, and intervention delivery was standardized through regular team meetings and protocol checklists to ensure consistency, appropriateness, and fidelity across participants.

### 2.5. Data Analysis

Statistical analyses were conducted using SPSS version 31 (IBM Corp., Armonk, NY, USA). Prior to analysis, data were reviewed for completeness, outliers, and normality, and missing data were handled appropriately. Statistical assumptions were verified before performing tests. Descriptive statistics, including frequencies, percentages, means, and standard deviations, were used to summarize sociodemographic characteristics and study variables. The Chi-square test assessed associations between categorical variables (e.g., demographic factors and patient activation levels). The independent-samples *t*-test compared mean patient activation scores before and after the intervention to determine its impact. Statistical significance was set at *p* < 0.05.

### 2.6. Ethical Considerations

Ethical approval was obtained from the Institutional Review Board (IRB) of University Medical City before study initiation (Approval No. E-24-9034). All data were anonymized and stored securely to preserve confidentiality. Informed consent was obtained from all participants, documenting voluntary participation and approval for the anonymized publication of their responses.

## 3. Results

### 3.1. Participants’ Characteristics

In total, 65 patients were enrolled in the study: 40 patients from the FMC, 18 from the chronic and diabetic clinic, and seven from the older adults’ care clinic. Across all FMC units, 33 patients were identified in Levels 1–2, while 32 were in Levels 3–4 and were monitored without intervention to observe activation changes over time (Table 1). Before the intervention, 28% of FMC patients were in Levels 1–2, which decreased to 25% after the intervention (Table 2). In the diabetic clinic, Levels 1–2 declined from 14% to 5%, and in the older adults’ care clinic, from 9% to 6%. Among all participants, 41.5% lived with diabetes or prediabetes, and 30.8% lived with two or more chronic conditions.

Activation declined over time among patients in Levels 3–4 who received no intervention. In the FMC, 69% began the study in Levels 3–4 but decreased to 56% after 6 months. In the older adults’ clinic, the proportion declined from 3% to 0%, and in the chronic and diabetic clinic, from 28% to 19% over the same period (Table 2).

### 3.2. Baseline Characteristics

The intervention group was slightly older than the control group (47.28 vs. 46.30, *p* > 0.05). No significant differences were found between groups in sex or age. Patient activation scores differed significantly at baseline (t = 8.53, *p* < 0.001). No significant baseline differences were observed in health outcomes, including cholesterol or systolic and diastolic blood pressure (Table 3).

### 3.3. Comparison Between the Groups at 6 Months

Independent-samples *t*-test results showed a statistically significant difference in mean activation score changes within 3 months after the intervention (Table 4). Activation increased significantly in the intervention group (*p* = 0.004), while activation declined in the control group over 6 months. No significant differences were observed between groups in clinical outcomes, including cholesterol or diastolic and systolic blood pressure.

## 4. Discussion

This study aimed to evaluate the effectiveness of a tailored patient activation intervention across diverse patient groups visiting an FMC. The findings indicate that structured follow-up and educational sessions can enhance patient activation, particularly among individuals with lower baseline activation. This pattern is consistent with the broader international literature demonstrating that activation-focused interventions are most associated with improvements among patients with initially low engagement and self-management capacity [25,26]. Foundational work by Hibbard et al. established that patient activation is a developmental construct, with greater potential for improvement among individuals at lower activation levels [5]. Similar trends have been reported in large observational and interventional studies from the USA and Europe: tailored coaching, follow-up, and education were associated with meaningful increases in activation among less activated patients, while changes among highly activated individuals were limited [27,28].

Our results align with findings from the Mayo Clinic, where the Adult Medical Care Coordination program improved patient activation compared with usual care. Similar to their study, significant improvements were observed among patients in PAM Levels 1 and 2, underscoring the value of targeted interventions for less activated patients [29].

However, unlike the Mayo Clinic findings, no improvement was observed in PAM Level 4 in our study. This difference may reflect the absence of tailored interventions for patients who were already highly activated, thereby limiting opportunities for measurable advancement [29]. Our results also align with Almutairi (2023), who reported improvements in PAM Levels 1 and 2 after intervention [19]. In contrast, we did not identify significant changes in clinical outcomes such as biometric or health service indicators. This finding is consistent with previous literature indicating that improvements in patient activation may not immediately translate into measurable clinical outcomes, particularly over short follow-up periods [7,13]. The lack of improvement in clinical outcomes observed in our study may be related to the relatively short study duration, which may not have been sufficient to capture the longer-term effects of increased patient activation on overall health status.

On the other hand, our results differ from those reported by Does et al. (2024) [21]. While their study found improvements in health outcomes without significant changes in activation, our findings showed the opposite pattern—gains in activation without corresponding improvements in clinical indicators [21]. This contrast highlights the complexity of the relationship between patient activation and health outcomes and suggests that increased activation may not yield measurable clinical benefits in the short term. Patients in the control group, who began at higher activation levels (Levels 3–4) and did not receive intervention, demonstrated a decline in activation over the study period. This pattern reinforces prior evidence that activation is dynamic and varies according to the level of support and engagement provided [13].

The absence of short-term improvements in clinical outcomes may partly be attributed to the broader construct of patient complexity, which refers to the combined influence of clinical, behavioral, socioeconomic, and system-level factors on health outcomes. Patient complexity frameworks indicate that outcomes are shaped by interacting burden and capacity dimensions, including multimorbidity, psychosocial needs, and healthcare demands, rather than by engagement alone [30,31]. A recent scoping review of complexity measures found that greater complexity is generally associated with longer hospital stays, reduced quality of life, and increased care needs, although the evidence remains heterogeneous and context dependent [32].

Improvements in patient activation are likely to strengthen individuals’ understanding, self-efficacy, and engagement in their care [7]. However, such improvements do not necessarily result in immediate changes in biometric indicators when underlying nursing and medical complexity remains substantial [33,34,35]. This interpretation offers a more plausible explanation for the pattern observed in this study, in which activation levels increased significantly despite the absence of short-term improvements in blood pressure, or cholesterol values.

Recent evidence indicates that specific nursing diagnoses—including impaired mobility, risk of injury, and acute pain—are closely associated with early clinical deterioration and an increased likelihood of transfer to the intensive care unit [35]. These findings suggest that patient outcomes are frequently shaped by the complexity of care needs and vulnerability profiles rather than by engagement alone. Within this context, the concept of patient activation should be viewed as an important enabling factor, but not as sufficient on their own to predict short-term clinical improvements.

Incorporating these perspectives may clarify why improvements in activation levels did not correlate with short-term biometric changes in this study. Future research should explicitly investigate how patterns of activation interact with medical and nursing complexity and assess whether models that combine these dimensions better predict enhanced clinical outcomes than activation alone.

### 4.1. Strengths and Limitations

This study has several limitations. First, the relatively small sample size restricts the statistical power of the analyses and limits the generalizability of the results to broader patient populations. Larger samples would allow for more robust subgroup analyses and a clearer understanding of how activation-based interventions perform across different demographic and clinical profiles. Second, the short follow-up period limited the ability to determine whether improvements in patient activation were sustained and whether they eventually translated into measurable changes in clinical outcomes, healthcare utilization, or long-term behavioral modifications. Longer-term follow-up studies are needed to evaluate the durability of these effects and to clarify the pathway linking increased activation to improved health status. Third, the absence of randomization limited causal inference; furthermore, baseline patient activation scores differed substantially between intervention and control groups, raising the possibility of regression to the mean.

Another limitation relates to the single-institution design, which may not fully reflect variations in care processes, resource availability, or patient engagement strategies across other hospitals or healthcare systems in Saudi Arabia. Institutional culture, staff training, and patient demographics may influence the outcomes and scalability of activation-based interventions. Additionally, self-reported activation data may be affected by response or social desirability bias, potentially leading to overestimation of post-intervention improvements. Despite these limitations, the study offers notable strengths. A key strength is the diversity of the recruited population, which included patients from the Diabetes and Chronic Diseases Unit, the Care of the Elderly Unit, and the General Family Medicine Unit. This heterogeneity allowed examination of the intervention’s adaptability across different clinical contexts and patient needs, enhancing the external relevance of the findings. Another important strength is the structured and replicable design of the intervention, which combined tailored educational sessions with follow-up strategies specifically developed for patients with lower activation levels (PAM Levels 1 and 2).

### 4.2. Study Implications

The findings of this study have several implications for clinical practice and healthcare system design. Integrating patient activation assessments, such as the PAM-13, into routine workflows offers clinicians a structured method for identifying each patient’s engagement level, readiness, and self-management capacity. Consistent with previous evidence, stratifying patients by activation level enables healthcare teams to tailor educational content, communication approaches, and follow-up intensity to individual needs, thereby supporting more efficient resource use and contributing to improved patient outcomes.

In chronic disease and diabetes care, where self-management is essential, activation-based approaches can strengthen patient confidence, improve adherence, and foster more collaborative clinician–patient relationships. In settings serving older adults, activation-based models must account for challenges such as multimorbidity, reduced health literacy, and functional limitations. Tailored interventions that incorporate family involvement, simplified educational resources, and consistent motivational reinforcement may enhance engagement and support the continuity of self-care behaviors despite these barriers.

Accordingly, activation-based interventions should be embedded within multidimensional care models that explicitly account for both nursing and medical complexity. In addition, future studies should examine combined models that integrate patient activation trajectories with measures of nursing complexity, such as the number and type of nursing diagnoses and the intensity of care. Evidence derived from such integrated approaches may better predict clinical deterioration and inform resource allocation than behavioral or clinical indicators alone.

Importantly, the decline observed among highly activated patients (PAM Level 4) illustrates that activation is dynamic and requires continuous support. Incorporating periodic PAM reassessment into follow-up visits can help clinicians identify early signs of disengagement and intervene promptly. From a systems perspective, integrating activation measures into electronic health records and quality improvement initiatives can strengthen data-driven decision-making, support benchmarking of engagement outcomes, and align care processes with value-based healthcare principles. Ultimately, adopting patient activation as a routine clinical metric may enhance the quality, efficiency, and patient-centeredness of care across diverse healthcare settings.

## 5. Conclusions

This study provides evidence that tailored interventions guided by the PAM-13 are feasible and are associated with the enhancement of patient activation within a tertiary care setting in Saudi Arabia. Consistent with earlier research, patients who received structured educational sessions and individualized follow-up demonstrated notable improvements in engagement, self-efficacy, and self-management behaviors. The greatest gains occurred among those with initially low activation (PAM Levels 1 and 2), emphasizing the importance of stratified, needs-based approaches to support patients in assuming a more active role in their health management. A progressive shift in activation levels was observed, with fewer patients remaining in lower PAM categories and more advancing to higher levels. These findings align with international evidence supporting the value of patient-centered strategies in improving adherence, motivation, and continuity of care. Despite these positive outcomes, the short study duration and single-center design limit generalizability. Further longitudinal and multicenter research is needed to evaluate the sustained effects of increased activation on clinical outcomes, healthcare utilization, and cost-effectiveness, particularly among patients with chronic diseases.

## Figures and Tables

**Table 1:** Patient Activation Measure (PAM) levels at baseline.

PAM Levels	n	%
Level 1 (Disengaged and Overwhelmed)	12	18.5
Level 2 (Becoming Aware but Still Struggling)	21	32.3
Level 3 (Tacking Actions)	21	32.3
Level 4 (Maintaining Behaviors and Pushing Forward)	11	16.9

**Table 2:** Patient Activation Measure (PAM) levels at baseline and follow-up by group.

Clinic	Group (n)	PAM Levels at Baseline (%)	PAM Levels at 6 Months (%)
Diabetic and chronic disease clinic	Intervention (32)	Level 1 & 2 (14%)	Level 1 & 2 (5%)
	Control (33)	Level 3 & 4 (28%)	Level 3 & 4 (19%)
Elderly care clinic	Intervention (32)	Level 1 & 2 (9%)	Level 1 & 2 (6%)
	Control (33)	Level 3 & 4 (3%)	Level 3 & 4 (0%)
Family medicine clinic	Intervention (32)	Level 1 & 2 (28%)	Level 1 & 2 (25%)
	Control (33)	Level 3 & 4 (69%)	Level 3 & 4 (56%)

**Table 3:** Baseline Scores.

Variables	Intervention (n = 32)	Control (n = 33)	*p*-Value
Mean Age (SD)	47.28 (14.66)	46.30 (13.59)	0.784
Sex			0.492
Female (%)	52.4	47.6	
Male (%)	43.5	56.5	
Mean Diastolic Blood Pressure Values (SD)	76.22 (15.66)	73.06 (9.61)	0.329
Mean Systolic Blood Pressure Values (SD)	130.22 (22.60)	161.06 (172.2)	0.319
Mean Cholesterol Values (SD)	4.86 (1.35)	4.61 (1.98)	0.473
Mean Patient Activation Scores (SD)	48.91 (5.14)	67.21 (11.02)	0.001

Independent *t*-test or chi-square test procedures were conducted to compare baseline differences in demographic variables between intervention and control groups.

**Table 4:** Comparison between Groups at 6 Months.

Variables	Intervention (n = 32)	Control (n = 33)	*p*-Value
Mean Diastolic Blood Pressure Values (SD)	71.77 (9.21)	73.03 (8.73)	0.581
Mean Systolic Blood Pressure Values (SD)	125.28 (22.78)	125.63 (15.05)	0.991
Mean Cholesterol Values (SD)	4.37 (0.94)	4.13 (0.93)	0.381
Mean Patient Activation Scores (SD)	53.92 (8.81)	61.22 (10.98)	0.004

Independent *t*-test procedures were conducted to compare baseline differences in the main study variables between intervention and control groups.

## Data Availability

The datasets generated and analyzed during the current study are not publicly available due to privacy and ethical restrictions but are available from the corresponding author upon reasonable request.

## Abbreviations

The following abbreviations are used in this manuscript:

ACO	Accountable Care Organization
FMC	Family Medicine Center
IRB	Institutional Review Board
PAM	Patient Activation Measure
PAM-13	13-item Patient Activation Measure
PAM-13-A	Arabic version of the 13-item Patient Activation Measure
SD	Standard Deviation
SPSS	Statistical Package for the Social Sciences
T2DM	Type 2 Diabetes Mellitus

## References

[1] Giusti A., Pukrittayakamee P., Alarja G., Farrant L., Hunter J., Mzimkulu O., Gwyther L., Williams N., Wannarit K., Abusalem L. (2022). Developing a global practice-based framework of person-centred care from primary data: A cross-national qualitative study with patients, caregivers and healthcare professionals. BMJ Glob. Health.

[2] Kwame A., Petrucka P.M. (2021). A literature-based study of patient-centered care and communication in nurse–patient interactions: Barriers, facilitators, and the way forward. BMC Nurs..

[3] Grover S., Fitzpatrick A., Azim F.T., Ariza-Vega P., Bellwood P., Burns J., Burton E., Fleig L., Clemson L., Hoppmann C.A. (2022). Defining and implementing patient-centered care: An umbrella review. Patient Educ. Couns..

[4] Alkhaibari R.A., Smith-Merry J., Forsyth R., Raymundo G.M. (2023). Patient-centered care in the Middle East and North African region: A systematic literature review. BMC Health Serv. Res..

[5] Hibbard J.H., Stockard J., Mahoney E.R., Tusler M. (2004). Development of the patient activation measure (PAM): Conceptualizing and measuring activation in patients and consumers. Health Serv. Res..

[6] Miller K.L. (2016). Patient centered care: A path to better health outcomes through engagement and activation. NeuroRehabilitation.

[7] Hibbard J.H., Greene J. (2013). What the evidence shows about patient activation: Better health outcomes and care experiences; fewer data on costs. Health Aff..

[8] Mosen D.M., Schmittdiel J., Hibbard J., Sobel D., Remmers C., Bellows J. (2023). Is patient activation associated with outcomes of care for adults with chronic conditions?. J. Ambul. Care Manag..

[9] Ivey S.L., Shortell S.M., Rodriguez H.P., Wang Y.E. (2018). Patient engagement in ACO practices and patient-reported outcomes among adults with co-occurring chronic disease and mental health conditions. Med. Care.

[10] Schuttner L., Reddy A., Rosland A.M., Nelson K., Wong E.S. (2020). Association of the implementation of the patient-centered medical home with quality of life in patients with multimorbidity. J. Gen. Intern. Med..

[11] Shortell S.M., Poon B.Y., Ramsay P.P., Rodriguez H.P., Ivey S.L., Huber T., Rich J., Summerfelt T. (2017). A multilevel analysis of patient engagement and patient-reported outcomes in primary care practices of accountable care organizations. J. Gen. Intern. Med..

[12] Greene J., Hibbard J.H. (2012). Why does patient activation matter? An examination of the relationships between patient activation and health-related outcomes. J. Gen. Intern. Med..

[13] Greene J., Hibbard J.H., Sacks R., Overton V., Parrotta C.D. (2015). When patient activation levels change, health outcomes and costs change, too. Health Aff..

[14] Hendriks M., Rademakers J. (2014). Relationships between patient activation, disease-specific knowledge and health outcomes among people with diabetes; a survey study. BMC Health Serv. Res..

[15] Lindsay A., Hibbard J.H., Boothroyd D.B., Glaseroff A., Asch S.M. (2018). Patient activation changes as a potential signal for changes in health care costs: Cohort study of US high-cost patients. J. Gen. Intern. Med..

[16] Lin M.-Y., Weng W.-S., Apriliyasari R.W., VAN Truong P., Tsai P.-S. (2020). Effects of patient activation intervention on chronic diseases: A meta-analysis. J. Nurs. Res..

[17] Krist A.H., Tong S.T., Aycock R.A., Longo D.R. (2017). Engaging patients in decision-making and behavior change to promote prevention. Stud. Health Technol. Inform..

[18] Albadrani M.S., Aljeelani Y.O., Farsi S.H., Aljohani M.A., Qarh A.A., Aljohani A.S., Alharbi A.A., Tobaiqi M.A.A., Aljohani A.M., Alzaman N.S. (2024). Effect of medication adherence on quality of life, activation measures, and health imagine in the elderly people: A cross-sectional study. BMC Geriatr..

[19] Almutairi N., Gopaldasani V., Hosseinzadeh H. (2023). The effect of a patient activation tailored intervention on type 2 diabetes self-management and clinical outcomes: A study from Saudi Arabian primary care settings. J. Diabetes Res..

[20] Candelaria D., Cacciata M., Serafica R., Reyes A.T., Lee J.A., Hildebrand J.A., Sta Maria A., Strömberg A., Evangelista L.S. (2025). Patient activation improves with a multi-component personalized mHealth intervention in older patients at risk of cardiovascular disease: A pilot randomized controlled trial. Eur. J. Cardiovasc. Nurs..

[21] Does M.B., Adams S.R., Kline-Simon A.H., Marino C., Charvat-Aguilar N., Weisner C.M., Rubinstein A.L., Ghadiali M., Cowan P., Young-Wolff K.C. (2024). A patient activation intervention in primary care for patients with chronic pain on long term opioid therapy: Results from a randomized control trial. BMC Health Serv. Res..

[22] Kearns R., Harris-Roxas B., McDonald J., Song H.J., Dennis S., Harris M. (2020). Implementing the Patient Activation Measure (PAM) in clinical settings for patients with chronic conditions: A scoping review. Integr. Healthc. J..

[23] Hibbard J.H., Mahoney E.R., Stockard J., Tusler M. (2005). Development and testing of a short form of the patient activation measure. Health Serv. Res..

[24] Cutilli C., Christensen S.A. (2024). Promoting health literacy. Am. Nurse J..

[25] Innab A., Kerari A. (2022). Impact of behavioral interventions on patient activation in adults with hypertension: A systematic review and meta-analysis. Inquiry.

[26] Innab A., Kerari A., Alqahtani N., Albloushi M., Alshammari A. (2023). Patient activation, adherence to hypertension treatment plans and blood pressure control in Saudi Arabia: A cross-sectional study. BMJ Open.

[27] Regeer H., van Empelen P., Bilo H.J.G., de Koning E.J.P., Huisman S.D. (2022). Change is possible: How increased patient activation is associated with favorable changes in well-being, self-management and health outcomes among people with type 2 diabetes mellitus: A prospective longitudinal study. Patient Educ. Couns..

[28] Abdelraheem O., Salama M., Chun S. (2024). Impact of digital interventions and online health communities in patient activation: Systematic review and meta-analysis. Int. J. Med. Inform..

[29] Savitz S.T., Lampman M.A., Inselman S.A., Hunt V.L., Mattson A.B., Stroebel R.J., McCabe P.J., Witwer S.G., Borah B.J. (2025). Impact of medical care coordination intervention on patient activation. Am. J. Manag. Care.

[30] Nicolaus S., Crelier B., Donzé J.D., Aubert C.E. (2022). Definition of patient complexity in adults: A narrative review. J. Multimorb. Comorb..

[31] Ben-Menahem S., Sialm A., Hachfeld A., Rauch A., von Krogh G., Furrer H. (2021). How do healthcare providers construe patient complexity? A qualitative study of multimorbidity in HIV outpatient clinical practice. BMJ Open.

[32] Kaneko H., Hanamoto A., Yamamoto-Kataoka S., Kataoka Y., Aoki T., Shirai K., Iso H. (2022). Evaluation of complexity measurement tools for correlations with health-related outcomes, health care costs and impacts on healthcare providers: A scoping review. Int. J. Environ. Res. Public Health.

[33] Lightfoot C.J., Nair D., Bennett P.N., Smith A.C., Griffin A.D., Warren M., Wilkinson T.J. (2022). Patient Activation: The Cornerstone of Effective Self-Management in Chronic Kidney Disease?. Kidney Dial..

[34] Sanson G., Welton J., Vellone E., Cocchieri A., Maurici M., Zega M., Alvaro R., D’Agostino F. (2019). Enhancing the performance of predictive models for Hospital mortality by adding nursing data. Int. J. Med. Inform..

[35] Cesare M., Nurchis M.C., Damiani G., Cocchieri A., Nursing and Public Health Group (2025). Ranking Nursing Diagnoses by Predictive Relevance for Intensive Care Unit Transfer Risk in Adult and Pediatric Patients: A Machine Learning Approach with Random Forest. Healthcare.

